# Reply to Palmirotta et al. Comment on “Kopańska et al. Disorders of the Cholinergic System in COVID-19 Era—A Review of the Latest Research. *Int. J. Mol. Sci.* 2022, *23*, 672”

**DOI:** 10.3390/ijms23052822

**Published:** 2022-03-04

**Authors:** Marta Kopańska, Jacek Szczygielski, Paulina Bartman, Agnieszka Banaś-Ząbczyk

**Affiliations:** 1 Department of Pathophysiology, Institute of Medical Sciences, Medical College of Rzeszow University, 35-959 Rzeszow, Poland; 2 Department of Neurosurgery, Institute of Medical Sciences, Medical College of Rzeszow University, 35-959 Rzeszow, Poland; jacek.szczygielski@vp.pl; 3 Department of Neurosurgery, Faculty of Medicine, Saarland University, 66-424 Homburg, Germany; 4 Students Science Club “Reh-Tech”, University of Rzeszow, 35-959 Rzeszow, Poland; bartman.paulina@wp.pl; 5 Department of Biology, Institute of Medical Sciences, Medical College of Rzeszow University, 35-959 Rzeszow, Poland; agnieszkabanas@o2.pl

We have carefully read the Letter to the Editor by Concetta Cafiero, Alessandra Micera, Agnese Re, Beniamino Schiavone, Giulio Benincasa, and Raffaele Palmirotta related to our paper entitled “Disorders of the Cholinergic System in COVID-19 Era—A Review of the Latest Research” [1] published in the International Journal of Molecular Science.

Thank you for your positive feedback and for providing us with topics that extend our article with new reports. In the commentary, the authors drew attention to other aspects of the issues raised in our review (e.g., paying attention to the treatment of elderly or young people with severe course of the disease [2]). We would like to take an opportunity to reply to the authors due to overlapping conclusions, and we also want to update some topics.

The authors conducted a study in which new regions of homology between neurotoxin and SARS-CoV-2 sequences have been identified [3]. Palmirotta et al. [3] noted that the presence of identical or functionally equivalent amino acids is not limited to the 375–390 region as previously described, but it is also present in other areas of the Spike glycoprotein, with a sequence homology of 47/86 aa. The authors also found homology for the Muscarinic toxin-like protein, identified in Bungarus multicinctus (Snake: Many-banded krait; UniPro tKB: Q9W727—3SO8_BUNMU), which exhibits an entirely similar sequence to Neurotoxin homolog NL1. Additionally, they found a homology between the cryptic epitope for the human antibody CR3022 and the 27aa Kappa-conotoxin-like as14a, identified in Conus cancellatus (Cancellate cone; Conus austini; UniPro tKB: P0C6S2—CLEA_CON-CF), with an overall sequence homology of 12/27 aa. Several of the conotoxin sequences that the authors searched in Uni Prot Knowledge Base (UniProtKB) database aligned with the SARS-CoV-2 Spike glycoprotein. The presented data indicate that regions of homology with neurotoxin-like peptides are more frequently and widely present on the Spike glycoprotein than previously suspected [3].

It is also appropriate to pay attention to the intestinal symptoms that arise as a result of COVID-19 infection, as the discovery of the action and penetration of the coronavirus into the gastrointestinal tract will allow for the preparation of new, better therapies targeting the gut microbiota. Inflammatory stimuli cause the release of cytokines and microbes that can cause dysbiosis, which results in the release of intestinal cytokines into the circulation (this increases systemic inflammation caused by COVID-19) [4]. Modulation of the gut microflora is believed to have the potential to alleviate COVID-19 and its related complications, but studies should be conducted to establish the safety and efficacy of probiotics. Once we discover the mechanisms involved in the entry of the coronavirus into the gastrointestinal tract (especially if we understand disease progression), it will be possible to design therapies based on the intestinal microbes, which will likely alleviate/reverse the effects of COVID-19 [5].

We also agree that SARS-CoV-2 has the ability to penetrate through the cholinergic receptors present in ocular tissues. The virus from infected tears can be bound by the conjunctiva, the cornea, or the epithelium of the nasolacrimal duct [6,7,8]. The virus binds to the ACE2 receptors mentioned by the authors, but there are also reports that SARS-CoV-2 may bind to the CD147 receptor (receptor cluster of differentiation 147). Previous studies have confirmed the presence of CD147 in both the tears and conjunctiva, corneal epithelium and endothelium, or in the retinal pigment epithelium. The presence of CD147 was also noticed in cells infected with SARS-CoV-2 [7].

In conclusion, we would like to emphasize once again how important it is to constantly update our knowledge about the cholinergic system and how it is affected by COVID-19. As one can see from both the authors’ comment and our reply, this is a rapidly evolving topic, so we encourage everyone to follow the latest research on this topic.
